# Activity pacing: moving beyond taking breaks and slowing down

**DOI:** 10.1007/s11136-018-1794-7

**Published:** 2018-02-02

**Authors:** Deborah Antcliff, Philip Keeley, Malcolm Campbell, Steve Woby, Anne-Maree Keenan, Linda McGowan

**Affiliations:** 1 Physiotherapy Department, Fairfield General Hospital, The Pennine Acute Hospitals NHS Trust, Rochdale Old Road, Bury, England BL9 7TD UK; 2 School of Healthcare, University of Leeds, Leeds, England UK; 3 Department of Health Sciences, University of Huddersfield, Huddersfield, England UK; 4 Division of Nursing, Midwifery and Social Work, School of Health Sciences, The University of Manchester, Manchester, England UK; 5 Research and Development Department, Salford Royal NHS Foundation Trust and The Pennine Acute Hospitals NHS Trust, Manchester, England UK

**Keywords:** Chronic pain, Chronic fatigue, Activity pacing, Acceptance, Physical activity

## Abstract

This brief communication responds to the paper by Jeong and Cho (Qual Life Res 26(4):903–911, 2017) that has described activity pacing in limited terms of adjusting activities through going at a slower rate and taking breaks. Activity pacing was reported as not involving goal setting, in comparison to other strategies for long-term conditions such as Acceptance and Commitment Therapy. This brief communication aims to challenge this limited perception of activity pacing in light of numerous studies that recognise pacing to be a more complex strategy. Pacing is considered to be a multifaceted coping strategy, including broad themes of not only adjusting activities, but also planning activities, having consistent activity levels, acceptance of current abilities and gradually increasing activities, and one that includes goal setting as a key facet. It is essential that pacing is both defined and measured as a multifaceted strategy in order to assess the outcomes of pacing, and for meaningful comparisons with other strategies regarding efficacy for the management of long-term conditions.

Activity pacing is a strategy that is frequently implemented to modify activities among patients with long-term conditions, for example, chronic pain and fatigue [1, 2]. The aims of activity pacing include to reduce overactivity–underactivity cycling (fluctuating between high and low levels of activity) in order to improve overall function and reduce the likelihood of exacerbating symptoms [3–5]. Activity pacing is considered to be a key component of pain management programmes and it is regarded as a facet of graded exercise therapy and cognitive behavioural therapy (CBT) [1, 6, 7].

With the development of cognitive therapies, there is an increasing use of third-wave therapies such as Acceptance and Commitment Therapy (ACT) in pain management programmes [8, 9]. ACT is underpinned by a process of psychological flexibility and it involves acceptance, value-based goals, committed action and cognitive defusion [10]. In contrast to CBT, ACT involves experiencing thoughts/emotions rather than modifying cognitions [10].

In the context of increasing evidence for the benefits of acceptance, and in particular, ACT in the management of persistent pain, Jeong and Cho [11] explored the associations between pain acceptance, physical activity and functioning in a cross-sectional questionnaire designed study. It was found that physical activity partially mediated the association between pain acceptance and physical functioning and psychological functioning, and that moderate physical activity and walking, but not vigorous activity were significantly associated with improved physical and psychological functioning.

The finding that moderate activity/walking was significantly associated with improved functioning was likened to the concept of activity pacing by Jeong and Cho [11], and pacing was defined in narrow terms of involving “either partially completing work or regulating work speed by taking breaks or slowing down” (p. 909), referencing Nielson et al. [12]. Since this 2001 publication by Nielson et al. [12], there has been discussion into the more complex nature of activity pacing and the likely multidimensionality of this concept [13–15]. Importantly, pacing is being described as a strategy that goes beyond just breaking down tasks/having rest breaks, and the concept of slowing down may not be globally endorsed as a facet of pacing [5, 16, 17]. In contrast, activity pacing may involve multiple facets of planning, prioritising, alternating activities and gradually increasing activities [13, 15, 17].

Jeong and Cho [11] state that the evidence for the efficacy of activity pacing on patients’ function is inconsistent, and reference studies that have utilised only unidimensional measures of activity pacing [12, 18]. These findings are compared to more favourable outcomes associated with committed action, and it is suggested that the difference in efficacy may be due to the utility of value-based goals in committed action. In comparison, Jeong and Cho [11] state that “activity pacing does not consider goals” (p. 909), with reference to our paper [16]. We disagree, since goal setting did reach consensus of inclusion as potential items for the activity pacing questionnaire (APQ) during the Delphi technique [16]. Moreover, when we refined the APQ to 26 items (APQ-26), there were two items referring to goal setting: “I set activity goals that were meaningful to me” and “I set activity goals that were realistic for me” [13]. Interestingly, these items loaded onto pacing subthemes within the APQ-26 labelled Activity planning and Activity acceptance, respectively.

Therefore, within the APQ-26 there are both facets of goal setting and a theme of acceptance of abilities. This concurs with many research papers that endorse goal setting as a facet of pacing [17, 19, 20], together with clinical practice that integrates concepts of pacing, acceptance, flexibility and setting meaningful goals. Although pacing is not considered to be part of ACT, it may share elements that are compatible with ACT [8]. We suggest that when activity pacing is instructed as a flexible strategy, and one that has the aim of increasing meaningful activities rather than directly aimed at reducing pain/symptoms, it complements principles of ACT.

It is essential to recognise that the nature and content of pacing varies across different approaches and measures of pacing. For example, adaptive pacing therapy may focus on slowing down, using rest breaks and conserving energy [21], and previous pacing scales may have measured dimensions that include an overall reduction in activity [12, 18, 22]. Furthermore, pacing may not involve goal setting when it is described as a symptom-contingent strategy, underpinned by energy conservation [2]. However, energy conservation/adaptive pacing therapy forms only one type of pacing. There are recommendations away from symptom-contingent pacing strategies and towards quota-contingent pacing in order to reduce symptom-led and potentially disabling behaviours [15, 17, 23]. The operant approach to pacing is driven by quota-contingent activities, and involves setting goals with a view to gradually increasing activity levels [15]. The APQ-26 aligns with an operant approach to pacing, which is reflected in the multifaceted content of the scale items and the five themes of pacing that emerged following factor analysis of the APQ-26. In addition to the aforementioned themes of Activity planning and Activity acceptance, the APQ-26 contains themes of Activity adjustment, Activity consistency and Activity progression [13].

Since pacing has been previously investigated when defined as adaptive pacing therapy [21], or measured using limited scales, the findings that pacing is ineffective or even associated with worsened function may begin to be explained. This restricted exploration into pacing may contribute to the potentially unjustified conclusions about pacing and abandonment of this concept in search for other methods. However, for many clinicians and researchers, pacing is an active rehabilitative therapy and one that is highly endorsed by patients. Consequently, we all need to ensure that activity pacing is comprehensively defined and measured as a multidimensional concept in future research to add clarity to the facets and effects of pacing.
